# Monthly Summary

**Published:** 1893-10

**Authors:** 


### Monthly Summary.

A SIMPLE HOLDFAST FOR PORCELAIN INLAYS.—By Dr. Genese, Baltimore, Md.—The following plan I find good : Get the impression of the cavity and edges with number 30 to 60 gold foil, when thoroughly fitted fill with hard wax, heat by spatula, and press to fit tightly. Remove and invest in Teague's compound as lightly as possible. When dry burn out the wax by laying the imprint wax on a piece of plaster to absorb the wax as melted; this leaves a true mold of the cavity and edges and no force to displace the mold. (No need to have the investment outside the gold overlap.) Now take some waste gold foil, roll it up to form a pellet that will lay inside the cavity about half the depth. Put the porcelain or glass mixture in quite wet, and press and dry with bibulous paper, then burn it in the usual way, firing each till perfectly formed and full enough. It should be a little below absolute fusing point or enough to thoroughly shrink the porcelain without vitrifying it till the last. layer. On removing the investment and peeling off the gold, the little pellet can be picked out, and a nice depression, with undercut edges, will be formed for the oxiphosphate to enter and firmly hold the inlay in place.—*Ohio Journal.*

---

THE USE OF COCAIN AS AN ANÆSTHETIC.—P. Reclus says subcutaneous injections of cocain in surgery are perfectly safe, and that accidents are only due to ignorance and carelessness.

As regards the strength of the solution, he severely criticised the use of strong solutions, limiting the strength to two per cent., preferring one per cent. in more extensive operations, in which ten to fifteen injections are necessary to anesthetize the region.

When the surgical operation is simple, as for instance the extirpation of a subcutaneous tumor, a sebaceous cyst of lipoma, Dr. Reclus, after having determined the place of the incision and its extent, thrusts the hypodermic needle through the thickness of the skin, pulling it back as soon as it has reached the cellular tissue. Then the rod of the piston of the syringe is pushed down till a slight white swelling becomes apparent on the skin. No more pain than that caused by the puncture should be inflicted on the patient, if the procedure is carried out properly.

When the injection is completed, a few minutes should elapse before beginning operation—three to four minutes when the solution is of two per cent. strength; five to six minutes when it is one per cent.—*Items of Interest.*

---

MY METHOD of constructing corners on dead incisors may not be new, but with me it has been so successful that I give it for the benefit of those who are looking for a better way of raising that class of teeth than the often unsuccessful method of "building up" with foil and the mallet.

After the roots have been treated, the apex successfully filled, and all soreness has passed away, cut away with a sand-paper disk, all uneven edges, till you have brought the enamel margin to a perfectly flat surface. Enlarge the canal for about two-thirds its length, and fit snugly a pin of fine gold, allowing it to project to the biting edge of the tooth. Remove the pin, take a piece of pure gold plate, a little larger than the surface of the cavity, lay it over the surface and drill a hole corresponding to the enlarged nerve canal, start the pin through the hole and drive to place. Remove pin and plate and solder together. Replace, and with a corundum wheel or engine, grind the plate even with the margin of the enamel. Build up the corner as you desire it when finished, with wax, remove, and insert in plaster and marble, dust the pin down. After the investment has thoroughly hardened, work out the wax and melt into the mold, formed by its 22 solder, "spatting" it down with a steel instrument before it cools. Before finishing, it may be well to try the piece in, and grind down any places on the surface which are not perfectly level with the enamel, finish, cement to place, and you have a corner which will not scale or pit, and will outlast any corner which can be built of foil. I have been trying this method in all such cases for five years, and have never had a failure to my knowledge. —*E. R. Vaughan.*

---

NITRATE OF SILVER.—By B. F. Arrington.—In applying nitrate of silver to cavities to arrest decay, or to prepare for painless excavating, I twirl cotton on a round-head bur drill, moisten the cotton with a saturated solution of the remedy, then touch the powder (scraped or crushed), and convey it to the cavity, rotate and keep in the cavity a few moments, then dry with a pellet of bibulous paper, and fill with beeswax to preclude moisture, till the following day, or for several days, if necessary. Around the necks of teeth near the gums, I apply the remedy in the same way, but for greater length of time, as I cannot, in such cases, use beeswax.

On the cutting edges, or grinding surface of the teeth, much worn away and sensitive, as is frequently seen, I apply the remedy in the stick form, direct to the sensitive surface for three to five seconds, which is ordinarily sufficient to abate all sensitiveness.

For removal of discoloration produced by application of nitrate of silver, a saturated solution of iodid potassium, stick and pulverized pumice or silex is all that is requisite.

To guard against the possibility of injury to mucous membrane by scraping particles of nitrate of silver, it is well to have at hand a strong solution of table salt. Quickly applied, it will prevent injury to the membrane.

I have never known injury to teeth by application of nitrate of silver, but results always satisfactory, as with sulphuric acid in treatment of pyorrhea alveolaris.

The man who first used it, and recommended use of it in treatment of teeth, possibly was buried a hundred years ago or more. There is no need now of questioning and disputing as to who first introduced the remedy. He is not here to assert his claim and receive thanks for the introduction of a good remedy.

To Dr. Stebbins, for resurrecting and infusing new life, thanks are due, and should be freely awarded by the profession at large.

The result is one of great power and merit. Give it a trial, watch results, and if satisfactory, say so. Use freely in practice, and recommend its use to others, and so do by all well indorsed and tested remedies, that prove valuable. That will be true conservatism, and *conservatism* must be the foundation stone of scientific, practical, common-sense practice of dentistry.—*Dental Surgery.*

---

REMARKABLE SURGICAL OPERATION.—Dr. Sutton, assisted by Drs. Stone, McGrew and Bomgardner, in the presence of many other physicians at the Allegheny Hospital of Allegheny, Pennsylvania, on the 26th of March, removed an ovarian tumor from a delicate woman, which weighed one hundred and twenty pounds, while the weight of the woman after the tumor was removed was only seventy-five pounds. The lady was forty-seven years of age, and was a private patient in the hospital, and for that reason her name is withheld.

When all was in readiness the patient was wheeled into the room and quickly placed on the table. Over the tumor, and about that line around the body, the measurement was five feet five inches, and over the tumor alone the measurement was thirty-six inches.

Dr. Sutton stood ready as Dr. McGrew administered the anesthetic, which was chloroform, instead of ether, as being more favorably adapted because less is required than of ether. The anesthetic performed its work rapidly, and then Dr. Sutton, assisted by Drs. Stone and Bomgardner, began the operation. The incision was made in a second, and it was soon found that the tumor was adherent to everything. The work was being performed with all possible haste, while the most careful watch was maintained over the patient. Contrary to expectation she held her advantage, and at 3.45, when the operation was completed, her condition was really better than had been anticipated. She recovered quickly from the effects of the anesthetic, and was fully conscious before being placed in bed.

The tumor was of four years' growth, and during the last two years extended almost as rapidly as a mushroom. For several months she has not been able to stand, and could lie down only with exceeding discomfort and severe suffering.

The operation was more than ordinarily interesting, because of the great size of the tumor, the patient's very low vitality at the time, and for weeks previously, and the quick and unlooked-for rally from the shock. At night her condition was regarded as very good, and while it is yet too soon to claim a successful issue the surgeons in charge have strong hopes.

This is the largest ovarian tumor ever taken from a living subject. It is probably nearly twice as large as the majority of ovarian tumors removed from living subjects. The only tumor of this kind ever removed that approaches this one in size, was taken from a dead body in the Edinburgh infirmary, and weighed one hundred and twelve pounds.

Since the above was set in type, the lady, Mrs. Brush, died, about thirty-six hours after the operation.—*D. & S. Microcosm.*

---

LYSOL.—Attention having been drawn by the recent cholera "scare" to the popularity of carbolic acid as a disinfectant, notice is being taken in medical circles of the even superior advantages for many purposes of the cresols as disinfectants. It was discovered that crude carbolic acid made soluble by the action of sulphuric acid surpassed in germicidal power an equally strong solution of pure phenol, besides with creolin, though free from carbolic acid, was proved to be of unmistakably superior disinfecting activity to the latter. Being insoluble in water, however, these cresols were neglected till the idea was hit on of combining them with rosin soap. Though very efficacious, these preparations were only emulsions; and it remained for the cresols to be made soluble, as now in the form of lysol, that what can be called the ideal soluble disinfectant should be made generally available. Lysol is produced by dissolving in fat, and subsequently saponifying, with the addition of alcohol, the fraction of tar oil which boils between 190° and 200° Cent. It is a brown, oily-looking, clear liquid, with a feebly aromatic creosote-like odor. It contains 50 per cent. of cresols, and it is miscible with water to a clear, saponaceous, frothing fluid. It acts, to all intents and purposes, as a soap; and it is admirably adapted for surgical operations.—*Items of Interest.*

---

WOMEN DENTISTS.—It is the intention of *The Dental Tribune* to devote considerable attention to the interest of women dentists. Probably few men are cognizant of the interest taken by women in our profession; from a list, which is as perfect as it can be made, but which by no means is complete, we find the number of women dentists in the United States to be as follows:

| | |
|---|---|
| California, - - - - | 6 |
| Colorado, - - - - | 4 |
| Connecticut, - - - - | 2 |
| District of Columbia, - - - | 3 |
| Florida, - - - - | 1 |
| Georgia, - - - - | 1 |
| Illinois, - - - - | 17 |
| Indiana, - - - - | 5 |
| Iowa, - - - - | 4 |
| Kansas, - - - - | 13 |
| Kentucky, - - - - | 1 |
| Maryland, - - - - | 1 |
| Massachusetts, - - - - | 2 |
| Michigan, - - - - | 6 |
| Minnesota, - - - - | |
| Mississippi, - - - - | 1 |
| Missouri, - - - - | 5 |

| | |
|---|---|
| Montana, | 3 |
| New Jersey, | 4 |
| New York, | 10 |
| Ohio, | 6 |
| Pennsylvania, | 30 |
| Rhode Island, | 2 |
| South Dakota, | 1 |
| Texas, | 6 |
| Utah, | 1 |
| Wisconsin, | 5 |

---

HOW I TREATED AN ABSCESS—By Dr. J. K. Moose.—Mr. C called on me last August to get relief from an aching tooth. On examination I found the right lower second bicuspid abscessed. I opened the cavity in the crown and washed the canal with warm water; then removed as much of dead nerve as I could. I then tried to force warm water through apical foramen, but failed.

I loaded my hypodermic syringe with a drop or two each of creosote and tr. iodin, passed the point of needle through open sinus, and along the tube till it came in contact with root of tooth; with some force, I emptied the contents of syringe into the abscess, letting the surplus run out and catching it on cotton.

With floss silk, saturated in creosote, I filled the nerve canal, and filled the crown with gutta-percha.

I saw the patient seven months after, and the abscess had disappeared; the sinus had healed; the gums looked natural and healthy, and the tooth had not given a moment's trouble. —*Southern Journal.*

---

RECENT experiments conducted at the Pasteur Institute, in Paris, have shown that drinking water may be completely freed of cholera bacilli by the addition of fifteen grains of citric acid to a quart of water. As citric acid is an acid of lemon juice, it would appear that strong lemonade would answer the purpose equally well.—*Good Health.*

